# Strategic solutions to overcome emerging public health challenges with emphasis on vector-borne diseases (VBDs)

**DOI:** 10.17179/excli2024-7695

**Published:** 2024-11-05

**Authors:** Milad Ahmadi Marzaleh, Marziyeh Hamyali Ainvand

**Affiliations:** 1 Department of Health in Disasters and Emergencies, School of Health Management and Information Sciences, Shiraz University of Medical Sciences, Shiraz, Iran

## ⁯

Nowadays, vector-borne diseases are emerging as global public health challenges (Dong and Soong, 2021[2]). Vector-borne diseases (VBDs) are infections where the disease-causing agents are transmitted by arthropod vectors such as mosquitoes, triatomine bugs, blackflies, tsetse flies, sandflies, lice, and ticks (Wilson et al., 2020[7]). Malaria, dengue, chikungunya, yellow fever, Zika, lymphatic filariasis, schistosomiasis, onchocerciasis, Chagas disease, leishmaniasis, and Japanese encephalitis are the major VBDs reported globally by the World Health Organization (GBD 2017 Causes of Death Collaborators, 2018[3]). These diseases threaten more than 80 % of the world's population and disproportionately affect poorer populations in tropical and subtropical regions. Other regionally important vector-borne diseases include African trypanosomiasis, Lyme disease, tick-borne encephalitis, and West Nile fever (Wilson et al., 2020[7]).

Among the vector-borne diseases, malaria is the leading killer, causing approximately 620,000 deaths in 2017 (mostly in Africa), followed by dengue fever with around 40,500 deaths (mainly in Asia). The estimated number of cases in 2017 was 209 million for malaria and 105 million for dengue. While most other neglected vector-borne diseases are not as lethal, they often result in chronic infections that can cause significant disability (GBD 2017 Causes of Death Collaborators, 2018[3]).

Various factors influence the emergence and spread of vector-borne diseases. Climate change and its consequences (global warming, precipitation, floods, droughts, storms, changes in land cover, ocean climate change, forest fires, heat waves, changes in sea levels) have become a serious threat to human health for the global community (Rocklöv and Dubrow, 2020[5]). This not only affects temperature, rainfall, and weather patterns, but climate change is also expected to adversely impact the burden of infectious diseases, including vector-borne diseases (Semenza et al., 2022[6]).

Climate change has now impacted the transmission and spread of vector-borne diseases, and its impacts are likely to become more severe (Pandey et al., 2021[4]). The consequences of climate change in high-risk areas can also lead to the failure of vector control programs and the emergence of explosive and widespread epidemics with high morbidity and mortality in diseases such as yellow fever, dengue fever, and Rift Valley fever among affected populations (Connolly, 2005[1]). Therefore, in such a situation, efforts to prevent and control vector-borne diseases must be intensified, and the duties and responsibilities of policymakers and scholars in this field must be increased (Pandey et al., 2021[4]).

Therefore, this text was written with the aim of providing global approaches to counter vector-borne diseases.

1. Using Genetically Modified Organisms (GMOs), males are sterilized and released into the environment to mate with females and do not produce offspring
2. Biological control, the use of predatory insects (mimic insects) to eliminate vector insects
3. Using pathogenic agents such as bacteria, fungi to block the transmission of malaria and other diseases
4. Integrated vector management (IVM), which uses a combination of different control methods (biological, chemical and genetic)
5. Introducing Wolbachia bacteria into mosquito populations and infecting them, which can reduce the transmission of diseases such as Dengue and Zika
6. Using satellite images and Geographic Information Systems (GIS) to monitor environmental factors that affect the habitats of vectors and the spread of disease
7. Applying climate change adaptation strategies, developing models to predict how climate change will affect vector populations and implementing these strategies
8. Integrating human, animal and environmental health strategies and using a one-health approach
9. Using big data and artificial intelligence technologies for forecasting modeling, predicting prevalence and optimizing intervention strategies.

Public health faces various challenges including climate change, disasters, emergencies, and the emergence and re-emergence of diseases. These trends have significantly impacted disease rates, especially infectious and vector-borne diseases, and have tested the preparedness of health systems. Addressing these challenges requires comprehensive and integrated approaches within communities and international cooperation to improve health system readiness, response, and resilience against future crises. Although there are effective interventions for many vector-borne diseases, the lack of resources hinders their effective control. Therefore, combining vector control programs to address multiple diseases simultaneously can be more cost-effective and lead to a reduction in these diseases. 

## Conflict of interest

None to declare.
